# Age‐associated expression of p21and p53 during human wound healing

**DOI:** 10.1111/acel.13354

**Published:** 2021-04-09

**Authors:** Chee W. Chia, Cheryl A. Sherman‐Baust, Sara A. Larson, Ritu Pandey, Roxanne Withers, Ajoy C. Karikkineth, Linda M. Zukley, Judith Campisi, Josephine M. Egan, Ranjan Sen, Luigi Ferrucci

**Affiliations:** ^1^ Laboratory of Clinical Investigation Intramural Research Program, National Institute on Aging, National Institutes of Health Baltimore MD USA; ^2^ Laboratory of Molecular Biology & Immunology Intramural Research Program National Institute on Aging, National Institutes of Health Baltimore MD USA; ^3^ Clinical Research Core & Biorepository Intramural Research Program National Institute on Aging, National Institutes of Health Baltimore MD USA; ^4^ Buck Institute for Research on Aging Novato CA USA; ^5^ Lawrence Berkeley National Laboratory Berkeley CA USA; ^6^ Translational Gerontology Branch Intramural Research Program National Institute on Aging, National Institutes of Health Baltimore MD USA

**Keywords:** aging, human wound healing, p21, p53

## Abstract

In mice, cellular senescence and senescence‐associated secretory phenotype (SASP) positively contribute to cutaneous wound healing. In this proof‐of‐concept study, we investigated the expressions of p16, p21, and other senescence‐associated biomarkers during human wound healing in 24 healthy subjects using a double‐biopsy experimental design. The first punch biopsy created the wound and established the baseline. The second biopsy, concentric to the first and taken several days after wounding, was used to probe for expression of biomarkers by immunohistochemistry and RNA FISH. To assess the effects of age, we recruited 12 sex‐matched younger (30.2 ± 1.3 years) and 12 sex‐matched older (75.6 ± 1.8 years) subjects. We found that p21 and p53, but not p16, were induced during healing in younger, but not older subjects. A role for Notch signaling in p21 expression was inferred from the inducible activation of *HES1*. Further, other SASP biomarkers such as dipeptidyl peptidase‐4 (DPP4) were significantly induced upon wounding in both younger and older groups, whereas matrix metallopeptidase 9 (MMP9) was induced only in the younger group. Senescence‐associated β‐galactosidase (SA‐β‐gal) was not detectable before or after wounding. This pilot study suggests the possibility that human cutaneous wound healing is characterized by differential expression of p21 and p53 between younger and older subjects.

## Graphical abstract

Wound healing in humans is characterized by significant induction of p21‐ and p53‐positive cells but not p16‐positive cells in younger individuals. Furthermore, DPP4, a SASP biomarker, was significantly induced on wounding in both younger and older individuals, whereas MMP9 was induced only in younger individuals.

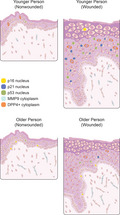

## 1 INTRODUCTION, RESULTS, AND DISCUSSION

Human skin wounds heal through four stages: platelet activation initiates a coagulation homeostatic cascade; inflammatory response, characterized by an influx of neutrophils, lymphocytes, and macrophages, follows and lasts 2–3 days; cell proliferation and formation of granulation tissue seal the damaged area over the next 2–4 weeks; remodeling and scar formation continue for months to complete the healing process (Enoch & Leaper, 2008). Wound healing is impaired in the elderly; however, molecular mechanisms that blunt wound healing with age have not been identified. Studies in murine models suggested that cellular senescence is important for wound healing (Demaria et al., 2014; Jun & Lau, 2010). Eliminating p16‐positive senescent cells delays wound healing, suggesting they contribute positively to the healing process (Demaria et al., 2014). Whether the same mechanism participates in human wound healing has not been evaluated. We designed a pilot study to probe the expression of senescence biomarkers in response to cutaneous wounds in healthy younger and older humans.

We studied two healthy cohorts: 12 younger (age: 30.2 ± 1.3 years; 6 men/6 women with age between 20 and 39 years) and 12 older (age: 75.6 ± 1.8 years; 6 men/6 women with age 70 years or older) subjects. At baseline (D_0_), we performed two 3 mm punch biopsies on non‐sun‐exposed skin of the inner upper arm, just below the axilla. At different days (D_x_) after the first visit, we performed two additional 6 mm punch biopsies concentric to the first biopsy (Figure S1). Different intervals from baseline to visit D_x_ were chosen to capture appearance of senescence biomarkers during human wound healing in the absence of prior knowledge. The protocol was approved by Institutional Review Board of the National Institutes of Health (Bethesda, MD). All participants consented to participate.

One of the two samples was fixed in formalin, dehydrated and embedded in paraffin, fixed and sectioned for immunohistochemistry (IHC) and RNA fluorescent in situ hybridization (RNA FISH). Photomicrographs were quantified by Indica Labs HALO v2.2.1870.31 software (see supplemental methods). Subjects returned for follow‐up visits at 6 (D_x+6_) and 16 days (D_x+16_) after the second biopsy for wound assessments. At 16 days after the second biopsy (D_x+16_), the wounds closed at a slower rate in the older compared to the younger group (*p* < 0.001), with no significant difference between men and women (Figure S2).

**FIGURE 1:**
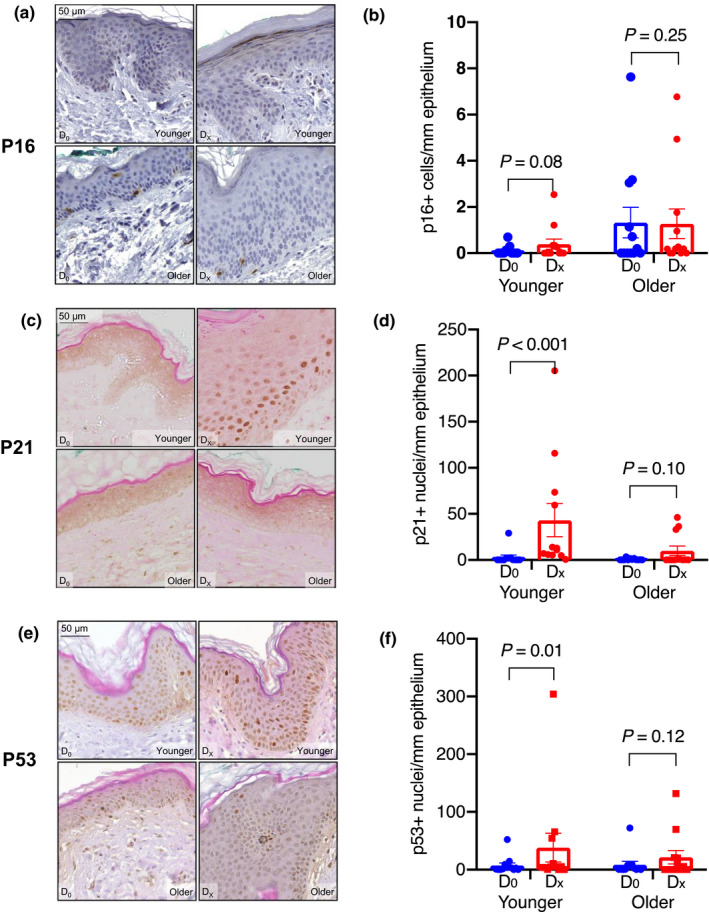
Representative images and quantification of p16, p21, and p53 proteins in normal skin at baseline (D_0_) and wounded skin (D_X_) (a,c,e). Brown staining in the nuclei indicates positivity for the indicated protein; expression was quantified as the number of positive cells per millimeter of epithelium (b,d,f). p16 expression was not significantly induced in response to wounding in either the younger or the older group (*p* = 0.08, *p* = 0.25) (b). p21 and p53 expressions were both significantly induced upon wounding in the younger (*p* < 0.001, *p* = 0.01), but not the older (*p* = 0.10, *p* = 0.12), groups (d,f). All images taken at 40× magnification

**FIGURE 2:**
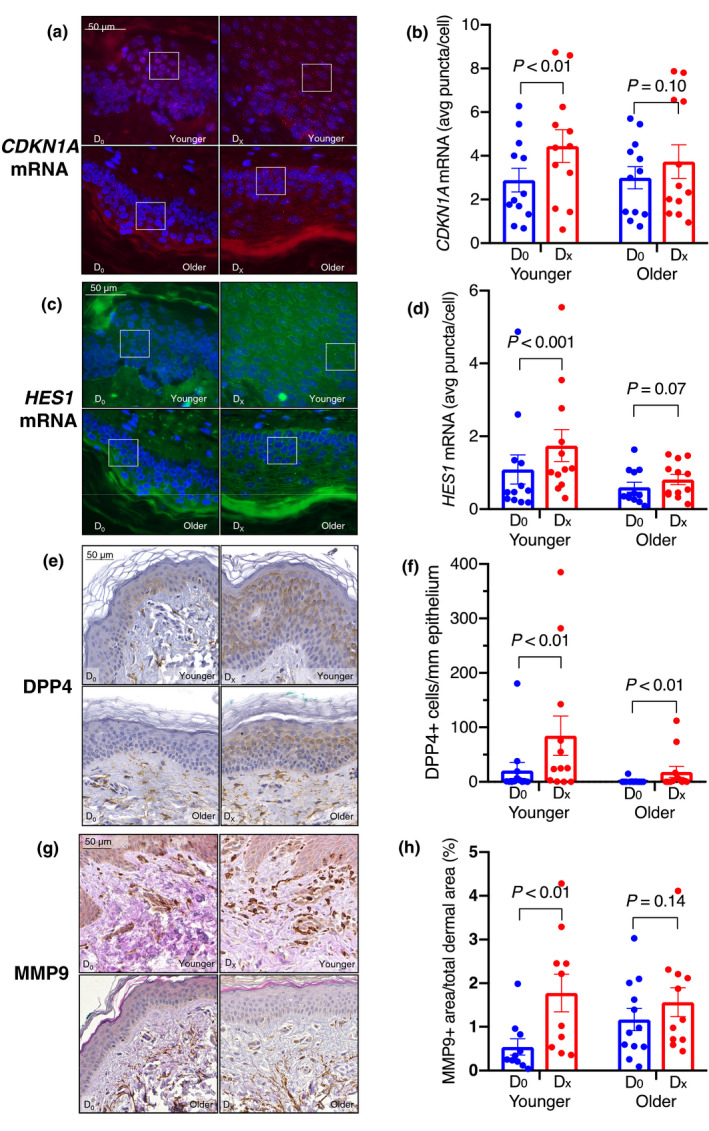
Representative images demonstrating *CDKN1A* mRNA (a), *HES1* mRNA (c), and immunohistochemistry of senescence markers DPP4 (e) and MMP9 (g) in normal skin at baseline (D_0_) and after wounding (D_X_). *CDKN1A* mRNA signal (pseudo‐colored red); *HES1* signal (pseudo‐colored green); DAPI (blue) as the nuclear stain. Each transcript is represented by a single punctum; the expression of each is represented as the average number of puncta per cell. Boxed areas are enlarged 2.5× in supplemental Figure S3. Both *CDKN1A* and *HES1* mRNA were induced in response to wounding in the younger (*p* < 0.01, *p* < 0.001), but not the older (*p* = 0.10, *p* = 0.07) group (b,d). Images in (a) and (c) are 63X magnification. DPP4 levels (number of positive cells per millimeter of epithelium) were significantly induced in wounded skin in both the younger and older group (*p* < 0.01 for both) (f). MMP9 levels (proportion of positive area in the papillary dermis), in response to wounding, were significantly higher in the younger (*p* < 0.01) but not older (*p* = 0.14) group (h). Images in (e) and (g) are 40× magnification

Baseline expression of p16 in epidermal cells (0.71 ± 0.35 cells/mm epidermis) was similar to previous reports (Waaijer et al., 2012, 2016). p21 and p53 were not significantly different between younger and older subjects at baseline (*p* = 0.41, *p* = 0.35, respectively). In response to wounding, we observed a modest but non‐significant increase in the number of p16‐expressing cells in younger (*p* = 0.08), but not older (*p* = 0.25) subjects (Figure 1a,b). By contrast, cells expressing p21 and p53 significantly increased in response to wounding in younger (*p* < 0.001 and *p* = 0.01), but not older (*p* = 0.10, *p* = 0.12) subjects (Figure 1c‐f). The increase in p21 protein was accompanied by a significant increase in *CDKN1A* mRNA, as measured by RNA FISH, in the younger subjects (Figure 2a,b).

Transcriptional activation of *CDKN1A* can occur through p53‐ and/or Notch‐driven pathways (Kreis et al., 2019). Indeed, there was significant positive correlation between the changes in p21 and p53 protein levels during wound healing (*r* = 0.61, *p* < 0.001). To probe for Notch activation, we assessed expression of the Notch target gene Hairy and Enhancer of Split 1 (*HES1*) by RNA FISH (Figure 2c). *HES1* mRNA levels increased significantly during wound healing in younger (*p* < 0.001), but not older (*p* = 0.07) subjects (Figure 2d). Changes in *CDKN1A* and *HES1* mRNA levels were also significantly correlated (*r* = 0.380, *p* = 0.03).

We evaluated the expression of two additional senescence‐associated biomarkers. DPP4 was recently identified as senescence marker in human diploid fibroblasts (Kim et al., 2017; Soare et al., 2020). DPP4 levels significantly increased, both in the membrane and cytoplasm of cells, during wound healing in both the younger and older subjects (*p* < 0.01 for both groups) (Figure 2e, f). We also examined MMP9 levels, a SASP component of human and mouse fibroblasts (Basisty et al., 2020; Demaria et al., 2017), and were quantified as the proportion of positive area in the papillary dermis. MMP9 levels significantly increased in response to wounding in younger (*p* < 0.01), but not older (*p* = 0.14), subjects (Figure 2g, h). Finally, we found no evidence of SA‐β‐gal activity in response to wounding in a subset of 9 subjects (Figure S4A).

We may have identified wound healing process features in humans that differ from those described in mice. In particular, we found that p21 and p53 but not p16 or SA‐B‐gal activity were induced during human wound healing. We surmise that p21 provides the required block to cellular proliferation that is required for keratinocyte differentiation after wound closure. The significant positive correlation between the changes in p21 and p53 protein levels, and that between the changes in *CDKN1A* and *HES1* mRNA, supports the view that p21 is induced by both p53‐ and Notch‐dependent mechanisms. This response to wound healing appears to entail activation of Notch. Interestingly, this response is possibly blunted in older persons for all markers examined except DPP4, providing a molecular clue for mechanisms involved in poor wound healing in the elderly and should be investigated in future studies. One major limitation of this pilot study is the small sample size, but the positive signals are encouraging and warrant further in‐depth studies.

The difference between mice and humans concerning induction of p16 needs further consideration. A possible interpretation is that the p21/p53 axis induces cellular senescence that is maintained by p16 (Beausejour et al., 2003; Stein et al., 1999). We cannot rule out the possibility that our sample size is too small or the observation period was too short to detect co‐expression of p16 and p21 as noted in mice (Demaria et al., 2014). Nor can we rule out the possibility that p16 dominates much later phases of human wound healing. Alternatively, our findings may reflect activation of the Notch pathway, rather than cellular senescence *per se*. Previous studies demonstrated that the Notch pathway is important in wound healing in mice (Chigurupati et al., 2007; Outtz et al., 2010) and declines with age (Conboy et al., 2003). Thus, an impairment of the Notch pathway may be the primary cause of retarder wound healing in older humans. Future studies should perform a more detailed and complete analysis of the Notch pathway.

## CONFLICT OF INTEREST

JC is a scientific founder of Unity Biotechnology. All other authors do not have any conflict of interest.

## AUTHOR CONTRIBUTIONS

CWC, RS, and LF conceptualized and designed the study. CWC, ACK, LMZ, and JME conducted the clinical study. CASB, SAL, RP, and RW conducted the IHC, RNA FISH, and imaging analyses. CWC, CASB, SAL, RP, RW, JC, JME, RS, and LF interpreted the data. CWC performed statistical analysis. CWC and CASB wrote the original draft of the manuscript. All authors contributed to review and editing of the manuscript.

## Supporting information

Supplementary Material

Supplementary Material

## Data Availability

The data that support the findings of this study are available from the corresponding author upon reasonable request.
