# Supplementary material for: Age‐associated expression of p21and p53 during human wound healing
Source: Aging Cell. 2021 Apr 9;20(5):e13354. doi: 10.1111/acel.13354 (PMC8135007; doi:10.1111/acel.13354)
Supplement: Supplementary file 1 — Supplementary Material [file ACEL-20-e13354-s002.pdf]

SUPPLEMENTAL METHODS

Study Design

We designed a proof-of-concept human study to determine whether p16, p21 and other senescence-associated biomarkers appear during wound healing in humans, similarly to what has been observed in mice. Subjects were recruited if they had no history of chronic diseases and were not taking medications that could affect wound healing. Subjects were stratified into two age groups – younger adults (age 20-39 years), and older adults (age ≥ 70 years). Eligible subjects had two 3mm skin biopsies on the inner upper-arm (non-sun exposed skin in the medial axilla) during the baseline visit (D_0) and two 6mm skin biopsies concentric to the previous site during a follow-up visit (D_x) at 6 different days (day 3, 5, 7, 9, 11 and 13) after the baseline visit (D_0) with one man and one woman from each age group for each specific day (Figure S1). Different intervals from baseline to visit D_x were aimed at capturing the appearance of p16- or p21-expressing cells during human wound healing in the absence of prior knowledge. After the 6mm biopsy (D_x), subjects returned for follow-up visit at 6 days (D_{x+6}) and 16 days (D_{x+16}) for wound assessments. Photographs of the wound sites were taken using a Nikon digital camera, with a standard wound measuring tape placed next to each wounds. The diameter of each wound was manually measured. Six of the 12 subjects in the younger group did not return for the final visit on day 16 (D_{x+16}).

Immunohistochemistry

p21, p53, DPP4 and MMP9

FFPE sections were deparaffinized, rehydrated, and heated to 96° C in 1X Target Retrieval Solution (Dako, S1700) for 20 minutes in a steamer. The sections were left to cool for 20 minutes at room temperature in retrieval solution. Immunohistochemistry (IHC) was performed using the UltraVision™ Quanto Detection System HRP DAB kit (Thermo Fisher, TL-060-QHD following the manufacturer's protocol). Antibodies and dilution information are listed in Table S1. The sections were incubated in DAB solution (1:33 dilution of chromogen to substrate). The DAB reaction was stopped by washing in deionized water. The sections were counterstained with hematoxylin, eosin or both, and then dehydrated and mounted in DPX (Dibutylphthalate Polystyrene Xylene).

p16

FFPE tissue sections were deparaffinized, rehydrated and then incubated in a steamer with preheated 1X epitope retrieval solution (Roche, 9517) at 95-99° C for 10 minutes. Sections were left to cool for 20 minutes at room temperature in the epitope retrieval solution. After washing sections in 1X wash buffer (Roche, 8557), IHC was performed using the CINtec® Histology Kit (Roche, 9517) according to supplier's protocol. The primary antibody was anti-p16 (Roche, 705-4793, ~1 mg/mL), and the negative control was monoclonal mouse anti-rat oxytocin-related neurophysin antibody, supplied with the kit (ready to use). Plastic coverslips were used during primary antibody and visualization reagent incubation to

prevent evaporation. After DAB incubation, sections were counterstained for two minutes in hematoxylin before being dehydrated and mounted in DPX.

RNA Fluorescent in Situ Hybridization (RNA FISH)

FFPE tissue sections were deparaffinized, rehydrated, and heated in citrate buffer at 96° C for 20 minutes in a steamer. Slides were briefly immersed in hot, deionized water before being washed in 100% ethanol and dried overnight at room temperature. RNA FISH was performed using Advanced Cell Diagnostics (ACD) RNAscope Fluorescent Multiplex Detection Reagents (320851). Using the HybEZ oven, tissues were permeabilized with protease III at 40° C for 30 minutes and then washed in deionized water. Tissues were incubated with target RNA-specific probes against p21 (ACD 311401-C3) and *HES1* (ACD 311191-C2) at 40° C for two hours. The first three amplification reagents were hybridized in succession by incubating at 40° C for 30 minutes and washing in wash buffer. The sections were incubated in the fluorophore for fifteen minutes before being washed and incubated in DAPI for one minute. After the DAPI was removed, the sections were mounted with Prolong Gold (Thermo Fischer, P36930) and left to cure for two nights at room temperature.

Senescence-associated β-galactosidase (SA-β-gal) Assay

Human diploid fibroblasts, IMR-90, were treated with 10Gy of ionizing radiation (IR) to induce senescence at day 7 (Casella et al., 2019; Nichols et al., 1977). Fresh frozen, 10uM skin biopsy sections from nine subjects, 7.5 month mutant APP/PS1 mouse brain (Borchelt et al., 1997; Zhang et al., 2019) sections and IR treated IMR-90 cells were fixed briefly in

paraformaldehyde and stained for SA-βGal activity overnight at 37°C, pH 6.0 using the Cell Signaling's Senescence β-Galactosidase Staining Kit (cat# 9860). Tissue sections were photographed with and without light H&E counterstain using a Leica microscope at 10X magnification. Our positive tissue control, mutant APP/PS1 mouse brain and human control, IR treated IMR-90 both showed abundant SA-b-gal activity (Figure S4b), as previously reported (Nichols et al., 1977; Zhang et al., 2019).

All antibodies were validated against reference tissues (Figure S5) and isotype (Figure S6).

Microscopy

All slides were imaged on the Zeiss Observer D1 at 40X using a Canon T1i (IHC) or AxioCam MRm at 40X (for quantitation), 63X (for publication) (RNA FISH), unless otherwise noted. All IHC whole tissue section images were captured using the Keyence BZ-X700 at 20X using an automatic tiling method and merged using the Keyence BZ-Analyzer program with no compression. Three representative images of each RNA FISH processed tissue were taken. Each image is a composite of the DAPI, p21 (Atto 647N),and *HES1* (Atto 550), captured independently. Before merging the images in HALO, we converted the images to grayscale in Photoshop CC 2018.

Image Analysis

All IHC images were quantitated using Indica Labs HALO v2.2.1870.31 software. The epithelium was annotated for targeted analysis. We used the image resolution μm/pixel to determine epithelium length and area in HALO. The Multiplex IHC module v1.2 was used to

analyze tissues stained for p21 and DPP4, and the Multiplex IHC module v2.1.1 was used to analyze tissues stained for p53 (Table S2). The number of DAB-positive nuclei per mm of epithelium (p21 and p53) or the number of DAB-positive cells per mm of epithelium (*DPP4*) was determined. p21 DAB-positive cells in sections with high background were counted manually. Area Quantification module v1.0 was used to analyze MMP9 signals in the papillary dermis of each tissue section for the proportion of MMP9 positive area (Table S3). We manually counted p16 positive cells across the entire epithelium. Epithelium length was determined using the curves option in the measure menu in Zeiss AxioVision V 4.8.2.0 software. Using the HALO FISH v2.1.7 module, the average number of HES1 and p21 puncta per cell and the average percent of Hes1+/p21+, Hes1+, and p21+ cells per image were determined in the annotated epithelium (Table S4). The average value and associated standard deviation of the three representative images for each tissue were calculated using Microsoft Excel Version 19.11.

Statistical Methods

After wounding on follow-up day (Dx), expression of all biomarkers within each cohort did not have age and healing time interaction or sex and healing time interaction; therefore, data were pooled into younger and older cohort for analyses (Figure S7). Results are reported as means ± SEM. An ANOVA approach was not used because in exploratory analyses, the data were not normally distributed. Two different nonparametric tests were used for analyzing within cohort and between cohort comparisons. Wilcoxon matched-pairs signed-rank test was used to compare the biomarkers before (D_0) and after wound induction (D_X) for within cohort comparisons. Mann-Whitney test was used to compare the relevant

biomarkers between younger cohort and older cohort. Linear mixed-effect model was used to determine the rate of clinical wound closure after D_X in both groups. Statistical calculations and graphic presentations were carried out using IBM SPSS Statistics Subscription (11-2018) and GraphPad Prism 8 for macOS, version 8.0.1 (GraphPad Software).

SUPPLEMENTAL REFERENCES

Borchelt, D. R., Ratovitski, T., van Lare, J., Lee, M. K., Gonzales, V., Jenkins, N. A., . . . Sisodia, S. S. (1997). Accelerated amyloid deposition in the brains of transgenic mice coexpressing mutant presenilin 1 and amyloid precursor proteins. *Neuron, 19*(4), 939-945. doi:10.1016/s0896-6273(00)80974-5

Casella, G., Munk, R., Kim, K. M., Piao, Y., De, S., Abdelmohsen, K., & Gorospe, M. (2019). Transcriptome signature of cellular senescence. *Nucleic Acids Res, 47*(21), 11476. doi:10.1093/nar/gkz879

Nichols, W. W., Murphy, D. G., Cristofalo, V. J., Toji, L. H., Greene, A. E., & Dwight, S. A. (1977). Characterization of a new human diploid cell strain, IMR-90. *Science, 196*(4285), 60-63. doi:10.1126/science.841339

Zhang, P., Kishimoto, Y., Grammatikakis, I., Gottimukkala, K., Cutler, R. G., Zhang, S., . . . Mattson, M. P. (2019). Senolytic therapy alleviates Aβ-associated oligodendrocyte progenitor cell senescence and cognitive deficits in an Alzheimer's disease model. *Nat Neurosci, 22*(5), 719-728. doi:10.1038/s41593-019-0372-9

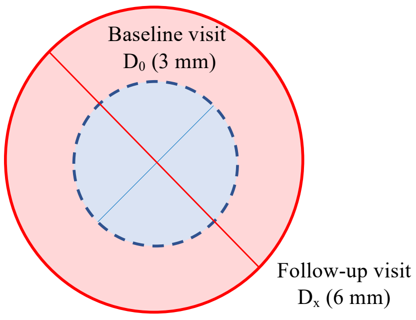

Figure S1. Size of the two concentric biopsies performed at baseline visit D_0 (3mm) and follow-up visit D_x (6mm).

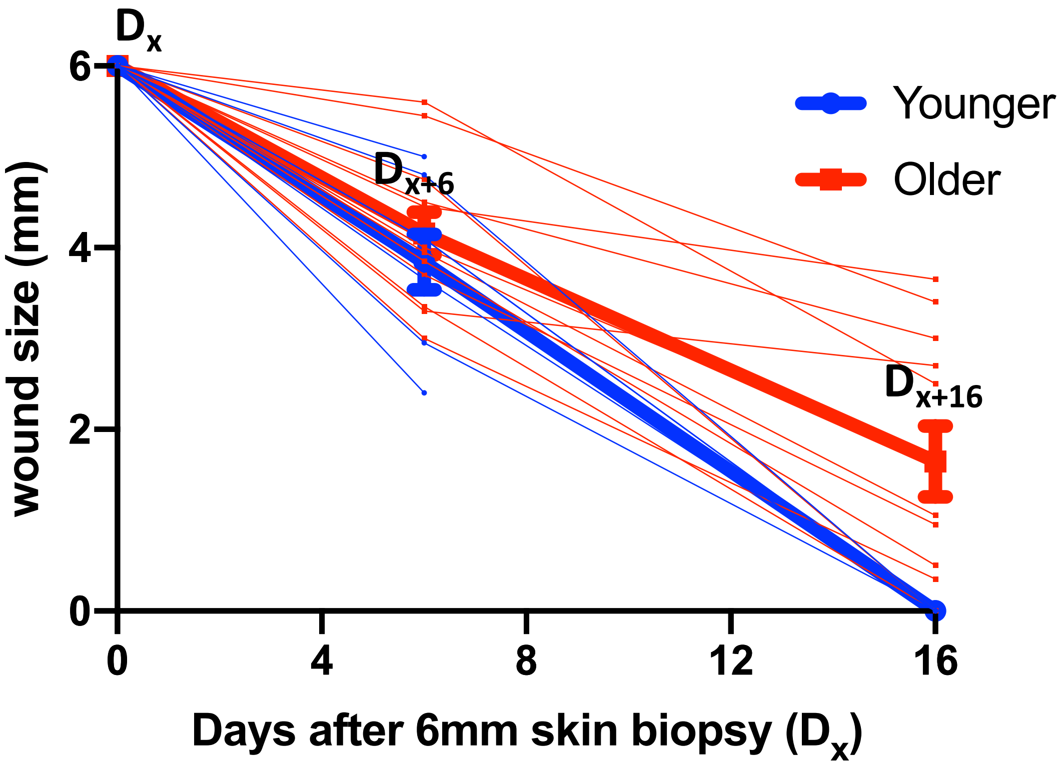

Figure S2. Rate of wound closure after 6mm biopsy (D_X). For day of 6mm biopsy (D_X) to 6 days after 6mm biopsy (D_{x+6}), the wound sites for the younger group closed at -0.36 mm/day compared to -0.31 mm/day for the older group. For day 6 (D_{x+6}) to day 16 (D_{x+16}) after 6mm biopsy, the wound sites for the younger group closed at -0.39 mm/day compared to -0.25 mm/day for the older group. The rate of wound closure was significantly different between the younger and the older group ($P <$ 0.001). Each thin line represents one subject. Thick line is the mean of all subjects for each group. Blue lines represent younger subjects and red lines represent older subjects. Six of the 12 subjects in the younger group did not return for the final visit on day 16 (D_{x+16}).

(a)

CDKN1A
2.5X digital
magnification
of original 63X
selected area

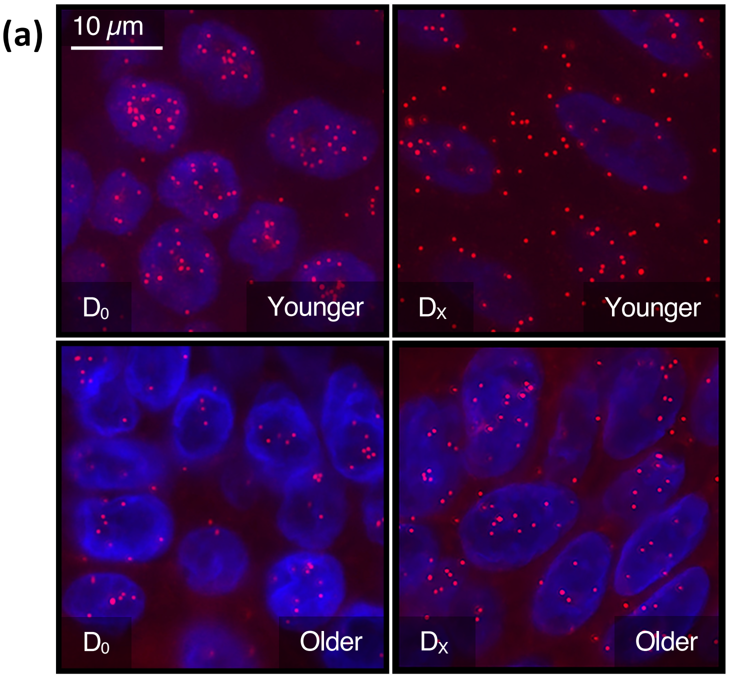

(b)

HES1
2.5X digital
magnification
of original 63X
selected area

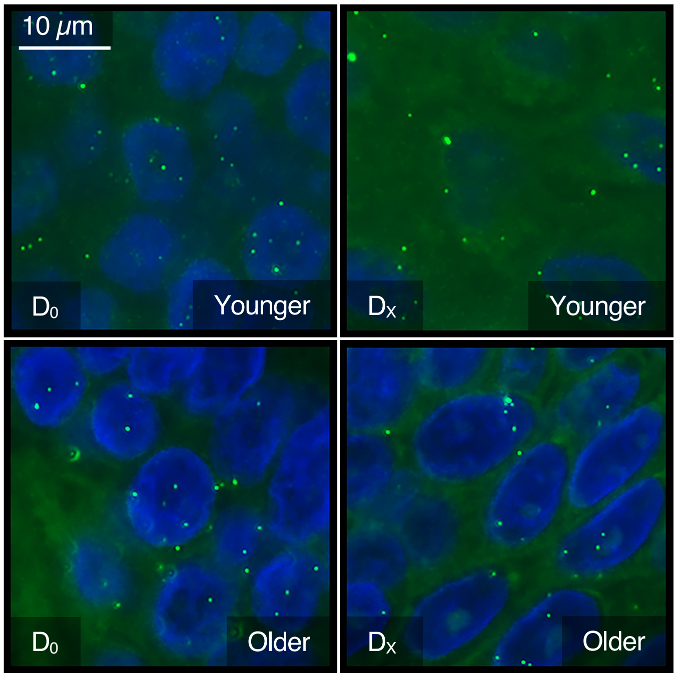

Figure S3. 2.5X digital magnification of selected areas of *CDKN1A* and *HES1* RNA FISH images in normal skin at baseline (D_0) and after wounding (D_X). Selected, boxed areas of Figure 2 RISH images are shown here, digitally enlarged 2.5X for better visualization of puncta. The *CDKN1A* mRNA signal was pseudo-colored red and *HES1* signal green, with DAPI (blue) as the nuclear stain. Each transcript is represented by a single punctum. Original images are 63X magnification.

Senescence-associated β-galactosidase Activity in normal skin at baseline (D_0) and after wounding (D_X).

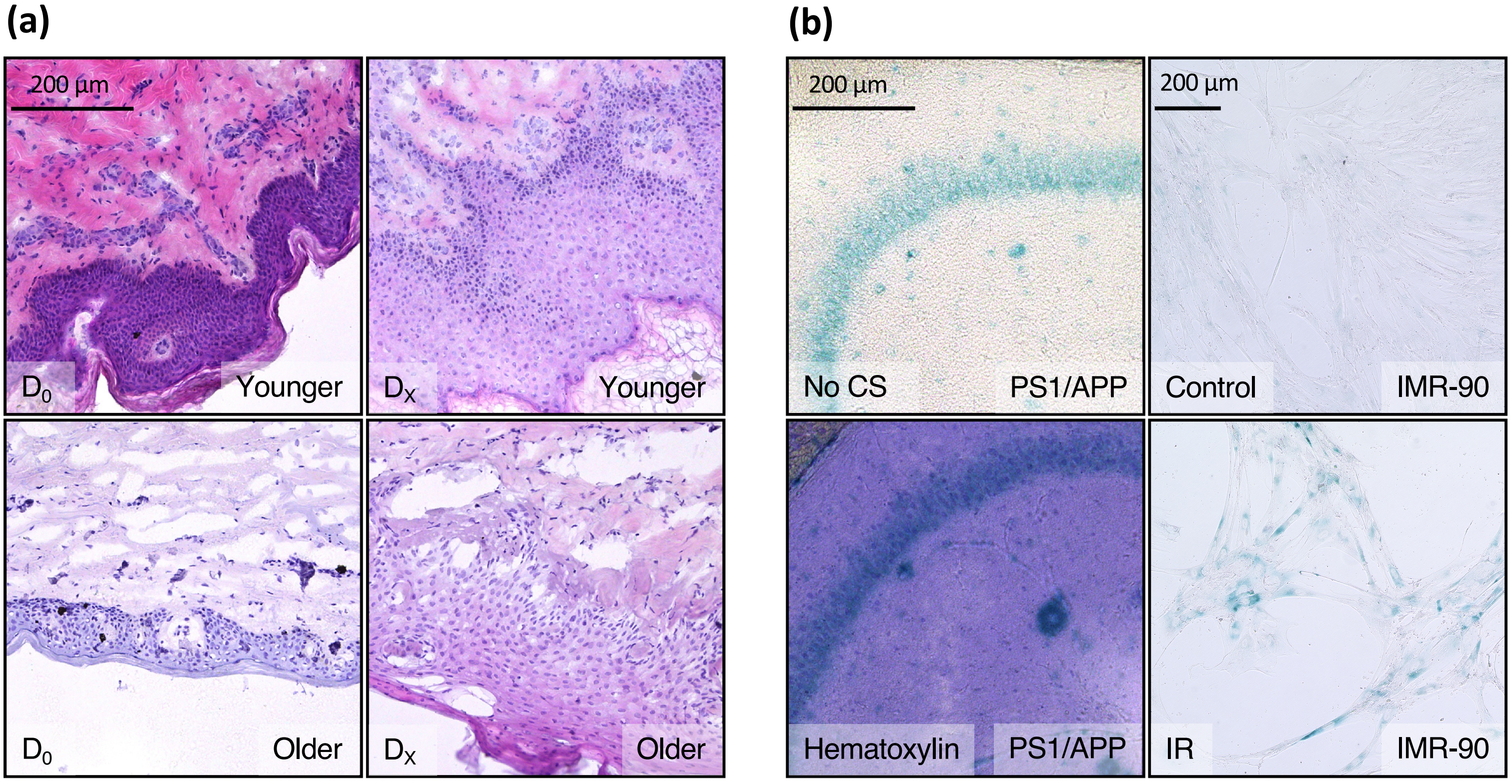

Figure S4. Senescence-associated β-galactosidase Activity in normal skin at baseline (D_0) and after wounding (D_X). Fresh, frozen, mounted skin biopsies of both younger and older participants at D_0 and D_X (a) and 2 sets of positive controls; mutant PS1/APP mouse brain and human IMR-90 untreated and 10 Gy γ IR cells (b) were assayed for senescence-associated β-galactosidase activity. Representative images are shown here at 10X magnification with 200 µm scale bars. β-gal activity was absent in the dermis and epidermis, at D_0 and D_X, regardless of age group (a). β-gal activity was found in both the PS1/APP mouse, with or without counterstain and in the IMR-90s 7 days past IR treatment, as evidenced by the blue-green signal (b).

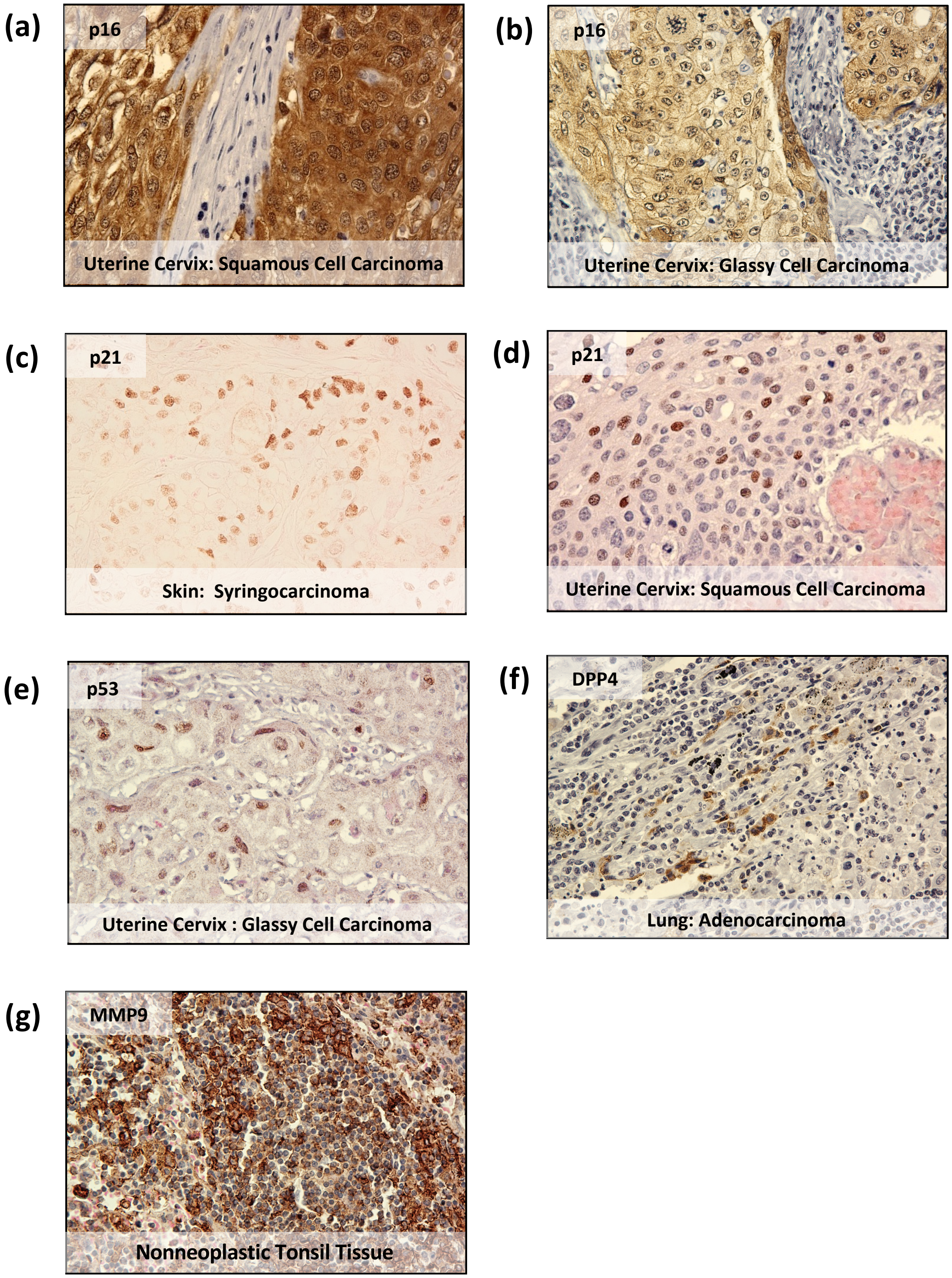

Figure S5. Positive (+) controls for all antibodies used. p16 (+) controls: Uterine cervical squamous cell and glassy cell carcinomas; counterstained with hematoxylin (a-b). p21 (+) controls: skin syringocarcinoma and uterine cervical squamous cell carcinoma; counterstained with eosin (c-d). p53 (+) control: uterine cervical glassy cell carcinoma; counterstained with hematoxylin and eosin (e). DPP4 (+) control: lung adenocarcinoma; counterstained with hematoxylin (f). MMP9 (+) control: nonneoplastic tonsil tissue; counterstained with hematoxylin and eosin (g).

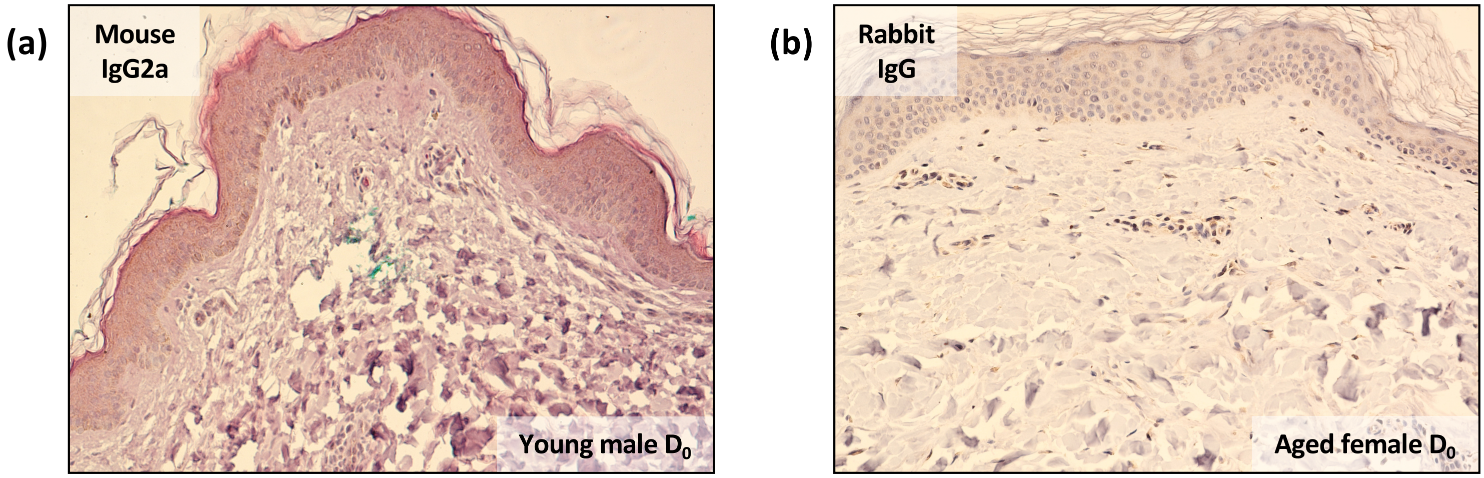

Figure S6. Isotype controls for all antibodies used. Isotype controls were used at an equal molar concentration to the antibody. **(a)** Mouse IgG2a used as an isotype control for anti-p53 and anti-p16 IHC. **(b)** Rabbit IgG used as an isotype control for anti-p21, anti-DPP4 and anti-MMP9 IHC.

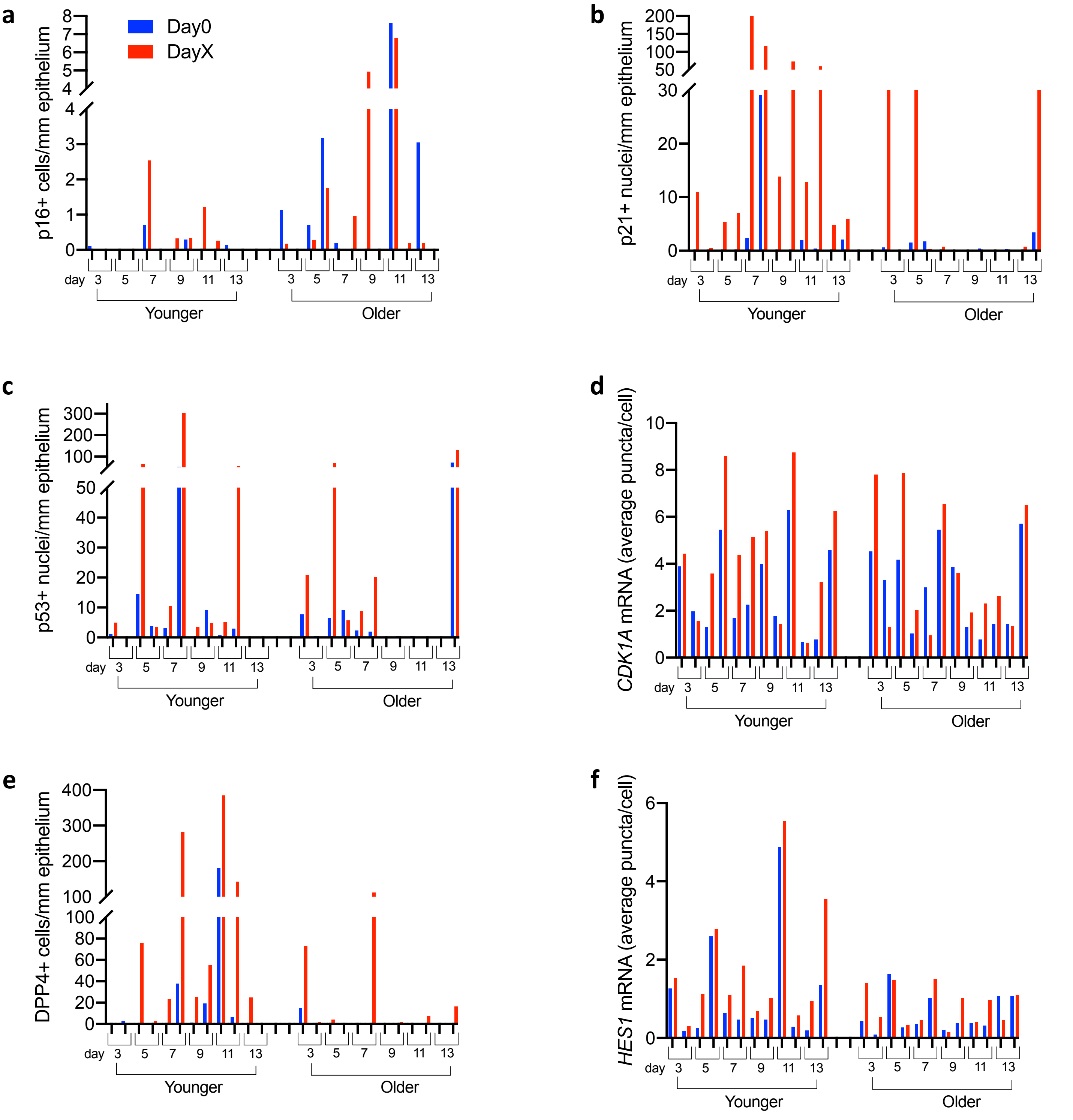

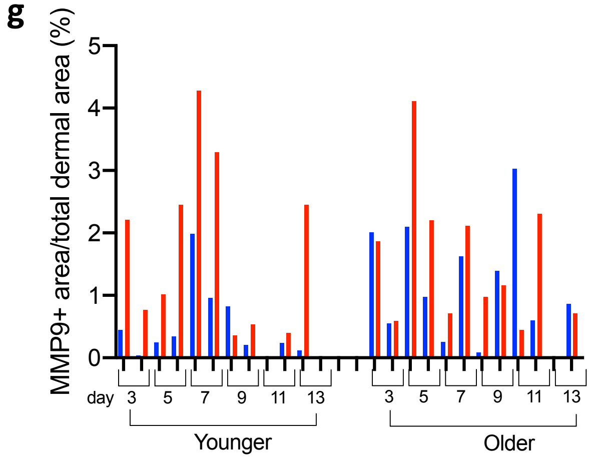

Figure S7: **p16, p21, p53, DPP4, and MMP9 protein levels (a,b,c,e,g) and *CDK1A* and *HES1* mRNA levels (d,f) before and after wounding for each subject**. Subjects are organized on the x axis by younger and older groups, and the day on which they were biopsied: baseline (Day_0) the visit 2 (Day_X). Protein levels are represented as the number of positive cells per millimeter of epithelium (a-c, e), or percentage of positive dermal area (g). mRNA levels are represented as the average number of puncta per cell (d,f).

Table S1: Primary antibody specifications and dilutions, and DAB incubation times

Protein	Primary antibody, Company, Catalog number	Dilution Factor	Isotype Control	DAB Incubation Time (mins:seconds)
p21	Abcam, ab109520	1:100	Rabbit IgG	3:30
p53	Santa Cruz Biotechnology, sc-126	1:400	Mouse IgG2a	16:00
p16	Roche, 705-4793	Pre-diluted	Pre-packaged in kit	10:00
DPP4	Cell Signaling, 40134	1:200	Rabbit IgG	3:30
MMP9	Abcam, ab76003	1:1000	Rabbit IgG	2:30

Table S2: HALO module Multiplex IHC v1.2 (p21, DPP4) and v2.1.1 (p53) settings

**HALO Parameters**	**p21**	**DPP4**	**p53**
Stain 1 (RGB settings)	DAB: 0.398, 0.794, 0.959	Hematoxylin: 0.357, 0.369, 0.200	Hematoxylin: 0.306, 0.335, 0.186
Stain 2 (RGB settings)	Eosin: 0.088, 0.381, 0.216	DAB: 0.361, 0.531, 0.708	Eosin: 0.128, 0.429, 0.179
Stain 3 (RGB settings)	-----	-----	DAB: 0.303, 0.600, 0.699
Misc. Stain (RGB settings)	Orientation Dye: 0.443, 0.211, 0.296	Orientation Dye: 0.580, 0.251, 0.357	-----
Stain 1 Nuclear Detection Weight (0-1)	0.351	0.377	0
Stain 2 Nuclear Detection Weight (0-1)	0	0	0
Stain 3 Nuclear Detection Weight (0-1)	-----	-----	1
Nuclear Contrast Threshold (0-1)	0.536	0.556	0.598
Minimum Nuclear OD (0-1)	0.225	0	0.225
Nuclear Size	$14.155 \leq x \leq 571.7$	$5.85 \leq x \leq 36.302$	$11.3 \leq x \leq 172.0274$
Minimum Nuclear Roundness	0.424	0.04	0.351
Nuclear Segmentation Aggressiveness	0.5	0.132	0.636
Fill Nuclear Holes	True	True	True
Maximum Cytoplasm Radius	0	6.75	0
Membrane Segmentation Aggressiveness	0.1	0	0
Cell Size	$9.8 \leq x \leq 571.7$	$9.8 \leq x \leq 561.947$	$10 \leq x \leq 600$
Stain 1 Membrane Segmentation	False	False	False
Stain 2 Membrane Segmentation	False	False	False
Stain 3 Membrane Segmentation	-----	-----	False
Stain 1 Nuclei Positive Threshold	0.4275	2.5	2.5
Stain 1 Cytoplasm Positive Threshold	2.5	2.5	2.5
Stain 1 Membrane Positive Threshold	2.5	2.5	2.5
Stain 1 Nucleus Mask	AND positive	-----	-----
Stain 2 Positive Nuclei Threshold	2.5	2.5	2.5
Stain 2 Positive Cytoplasm Threshold	2.5	0.1825	2.5
Stain 2 Positive Membrane Threshold	2.5	2.5	2.5
Stain 3 Nucleus Positive Threshold	-----	-----	0.417
Stain 3 Cytoplasm Positive Threshold	-----	-----	2.5
Stain 3 Membrane Positive Threshold	-----	-----	2.5

Table S3: HALO module Area Quantification v1.0 settings

**HALO Parameters**	MMP9
Stain 1 (RGB settings)	0.361, 0.744, 0.888 (DAB)
Stain 2 (RGB settings)	0.070, 0.331, 0.112 (Eosin)
Stain 3 (RGB settings)	0.154, 0.205, 0.139 (Hematoxylin)
Marker 1 Stain Min OD (0-1)	0.363, 0.363, 0.363
Marker 2 Stain Min OD (0-1)	1, 1, 1
Marker 3 Stain Min OD (0-1)	1, 1, 1

Table S4: HALO module FISH v2.1.7 settings

**HALO Parameters**	***CDKN1A* and *HES1***
Number of FISH probes	2
Number of Phenotypes	1
FISH Probe 1	HES1
HES1 Contrast Threshold	0.629
HES1 Signal Minimum Intensity	0.106
HES1 Spot Size	$0.1 \leq x \leq 0.4$
HES1 Copy Intenstiy	1
Spot Segmentation Aggressiveness	0.95
FISH Probe 2	CDKN1A
CDKN1A Contrast Threshold	0.53
CDKN1A Signal Minimum Intensity	0.232
CDKN1A Spot Size	$0.2 \leq x \leq 1$
CDKN1A Copy Intensity	1
Spot Segmentation Aggressiveness	1
Detect Cells	True
Nuclear Contrast Threshold	0.503
Minimum Nuclear Intensity	0.095
Nuclear Segmentation Aggressiveness	0.709
Fill Nuclear Holes	True
Nuclear Size	$11.3 \leq x \leq 160$
Minimum Nuclear Roundness	0.464
Minimum Cytoplasmic Radius	4
1+ Minimum Copies/Cell	1
2+ Minimum Copies/Cell	4
3+ Minimum Copies/Cell	10
4+ Minimum Copies/Cell	16
Phenotype 1 Name	HES1+/CDKN1A+
Phenotype1 Number of Criteria	2
Phenotype 1 Criteria 1 Filter	AND Positive
Phenotype 1 Criteria 2 Filter	AND Positive
