# Supplementary material for: Age‐associated expression of p21and p53 during human wound healing
Source: Aging Cell. 2021 Apr 9;20(5):e13354. doi: 10.1111/acel.13354 (PMC8135007; doi:10.1111/acel.13354)
Supplement: Supplementary file 2 — Supplementary Material [file ACEL-20-e13354-s001.docx]

**SUPPORTING INFORMATION**

Supplemental methods, figures and tables.

**Figure S1.** Size of the two concentric biopsies performed at baseline visit D_0_ (3mm) and follow-up visit D_x_ (6mm).

**Figure S2**. Rate of wound closure after 6mm biopsy (D_x_).

**Figure S3.** 2.5X digitaI magnification of selected areas of *CDKN1A* and *HES1* RNA FISH images in normal skin at baseline (D_0_) and after wounding (D_X_).

**Figure S4.** Senescence-associated β-galactosidase Activity in normal skin at baseline (D_0_) and after wounding (D_X_).

**Figure S5.** Positive (+) controls for all antibodies used.

**Figure S6.** Isotype controls for all antibodies used.

**Figure S7.** p16, p21, p53, DPP4, and MMP9 protein levels (a,b,c,e,g) and *CDK1A* and *HES1* mRNA levels (d,f) before and after wounding for each subject.

**Table S1.** Primary antibody specifications and dilutions, and DAB incubation times.

**Table S2.** HALO module Multiplex IHC v1.2 (p21, DPP4) and v2.1.1 (p53) settings

**Table S3.** HALO module Area Quantification v1.0 settings

**Table S4.** HALO module RNA FISH v2.1.7 settings
